# Neurodevelopmental risk and adaptation as a model for comorbidity among internalizing and externalizing disorders: genomics and cell-specific expression enriched morphometric study

**DOI:** 10.1186/s12916-023-02920-9

**Published:** 2023-08-04

**Authors:** Nanyu Kuang, Zhaowen Liu, Gechang Yu, Xinran Wu, Benjamin Becker, Huaxin Fan, Songjun Peng, Kai Zhang, Jiajia Zhao, Jujiao Kang, Guiying Dong, Xingming Zhao, Barbara J. Sahakian, Trevor W. Robbins, Wei Cheng, Jianfeng Feng, Gunter Schumann, Lena Palaniyappan, Jie Zhang

**Affiliations:** 1 Institute of Science and Technology for Brain Inspired Intelligence, Fudan University, Shanghai, People’s Republic of China; 2 Key Laboratory of Computational Neuroscience and Brain Inspired Intelligence, Ministry of Education, Fudan University, Beijing, People’s Republic of China; 3 School of Computer Science, Northwestern Polytechnical University, Xi’an, Shanxin People’s Republic of China; 4 Clinical Hospital of Chengdu Brain Science Institute, MOE Key Laboratory for Neuroinformation, University of Electronic Science and Technology of China, Chengdu, People’s Republic of China; 5 Institute of Computer Science and Technology, East China Normal University, Shanghai, People’s Republic of China; 6 Shanghai Center for Mathematical Sciences, Fudan University, Shanghai, People’s Republic of China; 7 MOE Key Laboratory of Computational Neuroscience and Brain-Inspired Intelligence and MOE Frontiers Center for Brain Science, Fudan University, Shanghai, People’s Republic of China; 8 Zhangjiang Fudan International Innovation Center, Shanghai, 200433 People’s Republic of China; 9 Department of Psychiatry, School of Clinical Medicine, University of Cambridge, Cambridge, UK; 10 Department of Psychology, Behavioural and Clinical Neuroscience Institute, University of Cambridge, Cambridge, UK; 11 Fudan ISTBI—ZJNU Algorithm Centre for Brain-inspired Intelligence, Zhejiang Normal University, Jinhua, 321004 China; 12 Shanghai Medical College and Zhongshan Hospital Immunotherapy Technology Transfer Center, Shanghai, 200032 China; 13 Shanghai Center for Mathematical Sciences, Shanghai, 200433 People’s Republic of China; 14 Department of Computer Science, University of Warwick, Coventry, CV4 7AL UK; 15 Collaborative Innovation Center for Brain Science, Fudan University, Shanghai, 200433 People’s Republic of China; 16 Fudan ISTBI—ZJNU Algorithm Centre for Brain-inspired Intelligence, Zhejiang Normal University, Jinhua, People’s Republic of China; 17 PONS Research Group, Department of Psychiatry and 20 Psychotherapy, Humboldt University, Berlin and Leibniz Institute for Neurobiology, Campus Charite Mitte, Magdeburg, Germany; 18 Douglas Mental Health University Institute, Department of Psychiatry, McGill University, Montreal, QC Canada; 19 Department of Psychiatry, Schulich School of Medicine and Dentistry, Western University, London, ON Canada; 20 Robarts Research Institute, University of Western Ontario, London, ON Canada; 21 Department of Medical Biophysica, Schulich School of Medicine and Dentistry, Western University, London, ON Canada

**Keywords:** Cortical surface area, Thickness, Developmental, Resilience, GWAS

## Abstract

**Background:**

Comorbidity is the rule rather than the exception for childhood and adolescent onset mental disorders, but we cannot predict its occurrence and do not know the neural mechanisms underlying comorbidity. We investigate if the effects of comorbid internalizing and externalizing disorders on anatomical differences represent a simple aggregate of the effects on each disorder and if these comorbidity-associated cortical surface differences relate to a distinct genetic underpinning.

**Methods:**

We studied the cortical surface area (SA) and thickness (CT) of 11,878 preadolescents (9–10 years) from the Adolescent Brain and Cognitive Development Study. Linear mixed models were implemented in comparative and association analyses among internalizing (dysthymia, major depressive disorder, disruptive mood dysregulation disorder, agoraphobia, panic disorder, specific phobia, separation anxiety disorder, social anxiety disorder, generalized anxiety disorder, post-traumatic stress disorder), externalizing (attention-deficit/hyperactivity disorder, oppositional defiant disorder, conduct disorder) diagnostic groups, a group with comorbidity of the two and a healthy control group. Genome-wide association analysis (GWAS) and cell type specificity analysis were performed on 4468 unrelated European participants from this cohort.

**Results:**

Smaller cortical surface area but higher thickness was noted across patient groups when compared to controls. Children with comorbid internalizing and externalizing disorders had more pronounced areal reduction than those without comorbidity, indicating an additive burden. In contrast, cortical thickness had a non-linear effect with comorbidity: the comorbid group had no significant CT differences, while those patient groups without comorbidity had significantly higher thickness compare to healthy controls. Distinct biological pathways were implicated in regional SA and CT differences. Specifically, CT differences were associated with immune-related processes implicating astrocytes and oligodendrocytes, while SA-related differences related mainly to inhibitory neurons.

**Conclusion:**

The emergence of comorbidity across distinct clusters of psychopathology is unlikely to be due to a simple additive neurobiological effect alone. Distinct developmental risk moderated by immune-related adaptation processes, with unique genetic and cell-specific factors, may contribute to underlying SA and CT differences. Children with the highest risk but lowest resilience, both captured in their developmental morphometry, may develop a comorbid illness pattern.

**Supplementary Information:**

The online version contains supplementary material available at 10.1186/s12916-023-02920-9.

## Background

Adolescence is a vulnerable period for gray matter maturation and many psychiatric disorders of adulthood begin at the preadolescent stage [1–3]. Preadolescent disorders can be broadly classified into internalizing and externalizing disorders, with a high degree of comorbidity between them [4]. For example, anxiety disorders (internalizing) are often comorbid with externalizing disorders such as attention deficit hyperactivity disorder (ADHD) [5–7] or conduct disorder (CD) [8], oppositional defiant disorder (ODD, externalizing) being comorbid with anxiety or depression (internalizing) [9]. This pattern is especially common in preadolescent period [10], during which the prevalence of comorbidity is greater than that of individual groups of disorders [11]. This pattern of comorbidity indicates a diminished response to conventional treatments as well as poor functional outcomes [12]. Furthermore, the pattern of comorbidity often emerges over time, and not identifiable at the outset, at the time of first presentation, precluding early interventions aimed at comorbidity. Despite this significant burden resulting from comorbidity, it is not clear if we can identify unique markers for comorbidity at the outset. We also do not know if comorbidity results from additive effect of disorder-specific mechanisms (shared) or arise from processes that are unique to the comorbid trajectory.

Transdiagnostic neuroimaging biomarkers have been identified, with the potential to track the vulnerability for psychiatric disorders even before overt clinical presentations occur [13, 14]. Two MRI-based markers of cortical morphology with distinct genetic basis and developmental trajectory [15, 16] are surface area (SA) and cortical thickness (CT). According to the radial unit hypothesis, the expansion of SA is driven by the proliferation of neural progenitor cells and tangential migration, while CT is related to the number of neurogenic divisions and radial migration [17]. Several studies indicate that internalizing and externalizing disorders have unique neurodevelopmental patterns reflected by their CT and SA alterations [18]. Some studies report opposing differences in CT in internalizing and externalizing disorders (e.g., anxiety relates to higher CT in the prefrontal cortex (PFC) and precentral gyrus [19]) while ADHD relates to reduced CT in the PFC and precentral regions [20].

A previous examination of the ABCD cohort found no association between general psychopathology (internalizing and externalizing symptoms) and CT. However, comorbidity was not specifically studied in this analysis [21]. In contrast, SA was found to be correlated with general psychopathology. This finding was validated in another independent cohort (mean age 10.6 years) [18]. Comorbidity of internalizing and externalizing disorders may have different morphometric correlates. On the one hand, CT and SA differences in comorbidity may be the result of additive influences of both disorders, with comorbid children exhibiting both patterns when compared to the healthy group. On the other hand, if a distinct rather than additive impairment results in comorbidity, we are more likely to see unique patterns of CT in comorbid cases (specific effect). Furthermore, CT and SA are under the influence of distinct sets of genes and biological processes [22]. Determining the unique contributions of CT and SA to comorbid internalizing and externalizing disorders could help uncover the developmental neurobiology of comorbidity. Ultimately, this may provide a reliable means for characterizing children who are likely to develop comorbidity for these two families of disorders.

In this study, we empirically test for the presence of additive (i.e., a simple aggregate effect) vs. unique morphometric patterns in children with internalizing and externalizing comorbidity using a large developmental cohort of preadolescent participants of the ABCD study [23]. While we remain agnostic as to the presence of additive vs. specific effects for comorbidity, we anticipate a divergence between CT and SA, given their discordant genetic and maturational trajectories [24–26]. Within this cohort, we selected a homogeneous group of unrelated European youth to perform a genome-wide association study (GWAS) and locate the genetic variants associated with regional SA and CT differences. This analysis was carried out in conjunction with a search for common genetic elements across the affected brain regions from the ABCD study and a determination of the brain cell type-specific expressions that shared maximum variance with the patterns of morphometric differences observed in the patient sample. Within internalizing/externalizing families of disorders, a high degree of overlap exists among individual disorders in terms of genetic heritability [27, 28] and neuroanatomical patterns [29]. As a result, we only consider comorbidity between the larger diagnostic families, i.e., between internalizing and externalizing disorders [30].

## Methods

### Definition of diagnostic families

**Fig. 1:**
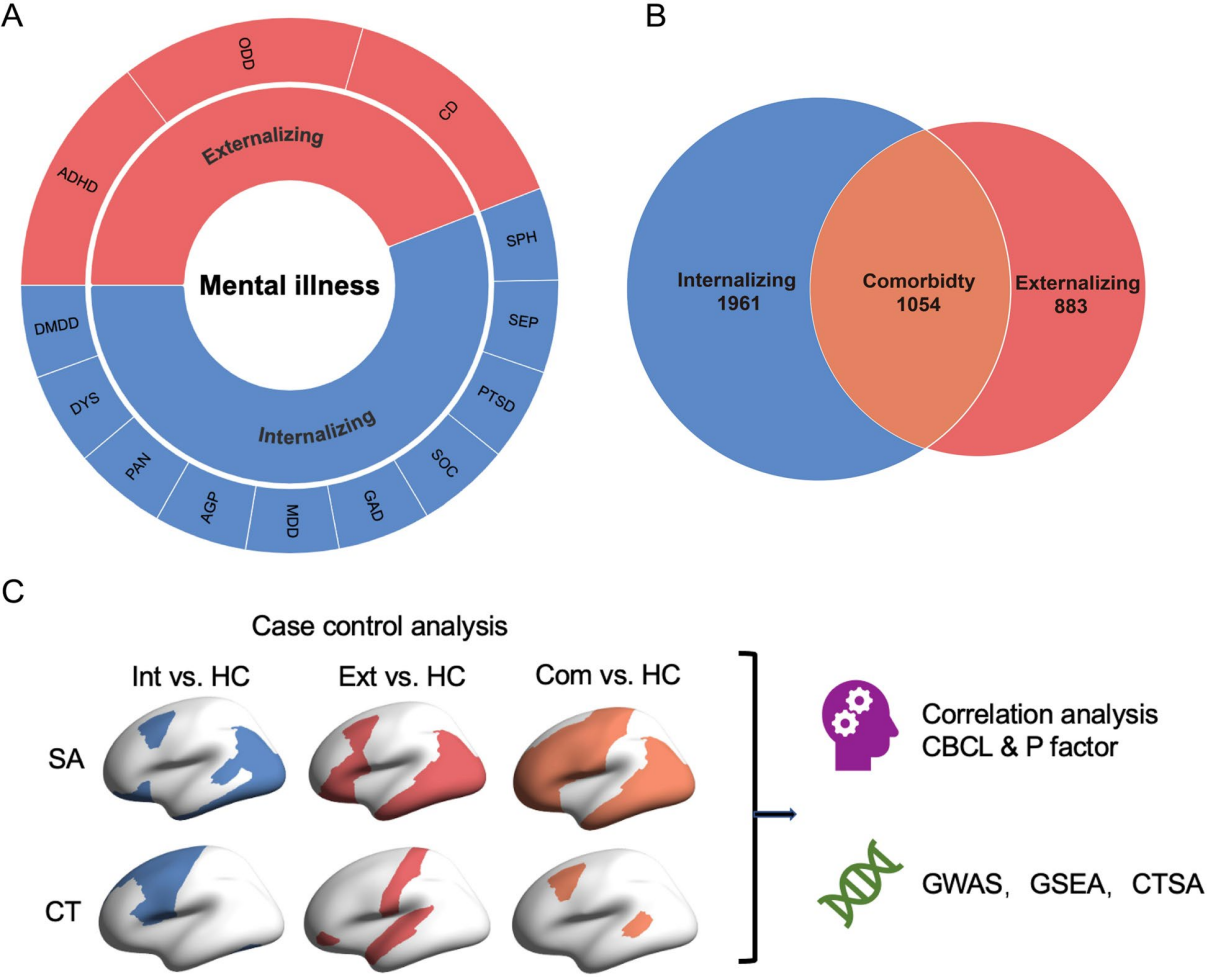
Components and comorbidity of externalizing and internalizing disorders. **A** Thirteen mental disorders (outer circle) were classified into two transdiagnostic categories (inner circle), i.e., externalizing and internalizing disorders. **B** Venn diagram depicting the overlap between the 2 transdiagnostic categories. Pure subsets of two transdiagnostic categories: externalizing disorder, red; internalizing disorder, blue. Comorbid between internalizing and externalizing disorders, orange. Children with thought disorder have been eliminated from membership of either external disorder or internal disorder. **C** An overview of all analysis. *Abbreviations*: ADHD, attention deficit hyperactivity disorder; CD, conduct disorder; ODD, oppositional defiant disorder; MDD, major depressive disorder; GAD, generalized anxiety disorder; SOC, social anxiety disorder; SEP, separation anxiety disorder; PTSD, post-traumatic stress disorder; AGP, agoraphobia; SPH, specific phobia; PAN, panic disorder; DYS, dysthymia; DMDD, disruptive mood dysregulation disorder; DEL, delusions; SA, surface area; CT, cortical thickness; Int, internalizing disorders; Ext, externalizing disorders; Com, comorbid between internalizing and externalizing disorders; HC, healthy control; CBCL, Child Behavior Checklist; GWAS, genome-wide association study; GSEA, gene set enrichment analysis; CTSA, cell type specificity analysis

Mental disorder diagnoses were determined by using parent or guardian responses to the computerized Kiddie Schedule for Affective Disorders and Schizophrenia (KSADS) based on the Diagnostic and Statistical Manual of Mental Disorders, fifth edition (DSM-5) criteria [31]. Lifetime (past or present) diagnoses of the 18 disorders were used [21]. Based on the definition of broad diagnostic families adopted in recent studies [32], two broad diagnostic families including externalizing disorders (attention-deficit/hyperactivity disorder, oppositional defiant disorder, conduct disorder), internalizing disorders (dysthymia, major depressive disorder, disruptive mood dysregulation disorder, agoraphobia, panic disorder, specific phobia, separation anxiety disorder, social anxiety disorder, generalized anxiety disorder, post-traumatic stress disorder) were used in our analysis (Fig. 1A). We excluded children with thought disorders (hallucinations, delusions, associated psychotic symptoms, bipolar disorder, obsessive–compulsive disorder) in the manuscript for two reasons: (1) the sample size of thought disorders is too small (*N* = 347) compared to that of externalizing/internalizing disorders, and (2) involving thought disorders would make the single diagnostic families (externalizing or internalizing) contain children with comorbidity. We also consider the influence of thought disorders in Additional file 1 [33–54].

We consider 3 broader diagnostic groups: pure internalizing and externalizing disorders and their comorbidity (see Fig. 1B). Healthy control preadolescents were those who did not have any mental disorders diagnoses (including unspecified or other specified disorders, eating disorders, alcohol use disorder, substance-related disorder, sleep problems, suicidal ideation or behavior, and homicidal ideation or behavior).

### Participants

Preadolescents aged 9–10 years (*N* = 11,878) are recruited from 22 research sites across the USA from the Adolescent Brain Cognitive Development (ABCD) Study® (release 3.0, November 2020), which contains physical and mental health, cognition, genetic, and neuroimaging data. The ABCD study group obtained written and oral informed consent from parents and children, respectively. Lifetime psychiatric diagnoses were determined using K-SADS-5. Demographic data of the ABCD sample was listed in Additional file 2: Table S1 and Table S2. We consider four groups: externalizing, internalizing, comorbidities between internalizing and externalizing disorders, and healthy control groups.

### Structural image acquisition and quality control

T1-weighted structural MRI data were gathered on 3-T MRI systems (Siemens Prisma, General Electric MR 750, Philips). On the basis of standardized processing pipelines [23], structural MRI data processing was collected using FreeSurfer version 5.3.070. All scan sessions completed radiological review whereby scans with incidental results were identified. Participants were removed who could not pass the visual inspection of T1 images and FreeSurfer quality control [55] (imgincl_t1w_include =  = 1). According to the Desikan-Killiany Atlas, the current analysis used post-processed SA and CT data which were mapped to 34 cortical parcellations per hemisphere (68 brain regions in total) [56].

### Child Behavior Checklist (CBCL)

The Child Behavior Checklist (CBCL), collected by the child’s caregiver or parents, is generally used to measure emotional and behavioral problems for children. The resulting scores used in ABCD include eight syndrome scale scores (anxious/depressed, withdrawn/depressed, somatic complaints, social problems, thought problems, attention problems, rule-breaking behavior, aggressive behavior), three summary scores (internalizing problems, externalizing problems, and total problems), six DSM-oriented scale scores (depressive problems, anxiety problems, somatic problems, attention deficit/hyperactivity problems, oppositional defiant problems, and conduct problems), and three 2007 scale scores (sluggish cognitive tempo, obsessive–compulsive problems, and stress problems). In the current analyses, we used raw scores of 16 CBCL scales from the baseline (*N* = 11,878) including anxious/depressed, withdrawn/depressed, somatic complaints, social problems, attention problems, rule-breaking behavior, aggressive behavior, depressive problems, anxiety problems, somatic problems, attention deficit/hyperactivity problems, oppositional defiant problems, conduct problems, sluggish cognitive tempo, obsessive–compulsive problems, and stress problems.

### Case–control analysis and ANOVA

We employed linear mixed models (LMM) with MATLAB (R2018b) to estimate the difference in CT and SA among each of the three transdiagnostic groups (externalizing, internalizing, and comorbid) to the healthy children group. Our LMM included random effects for family nested within the acquisition site. At the same time, it included fixed-effect covariates for sex, age, race/ethnicity (White, Hispanic, Black, Asian, others/mixed), pubertal status, parental marital status, total intracranial volume, parental education, and body max index (BMI). We did not adjust the global metrics (mean CT or total SA) in the analyses as it could attenuate or obscure the specific effects and contributions of regional brain structure. All analyses were false discovery rate (FDR, *q* = 0.05) corrected for multiple comparisons. We also implemented an analysis of variance (ANOVA) to examine the difference in CT and SA among externalizing, internalizing disorders, comorbidity, and healthy preadolescents after regressing out the same covariates using LMM. Tukey test was also performed among four groups in post hoc analysis.

### Correlation with symptoms

A general psychopathology factor (p-factor) and three sub-factors, externalized disorder (Ext), internalized disorder (Int), and thought disorder (Tho) were modeled using the parent-rated K-SADS-5 [57, 58]. Based on a prior observation from the ABCD study [35], employing a hierarchical model of externalizing (ADHD, ODD, CD), internalizing (MDD, GAD, PTSD, PD, SEP, SAD), thought (hallucinations, delusions, OCD, BP) disorder scores, we derived the p-factor using confirmatory factor analysis (R v4.0, *cfa* function of the *lavaan* package). This analysis was based on the whole sample (*N* = 11,878). We also performed association analyses between the total CBCL scores and the morphometric variables SA extracted from regions affected in the comorbid group compared to healthy subjects. For the CBCL symptom correlations, all children with symptom scores (*N* = 7570) were included irrespective of the diagnostic classifications, after regressing out the same covariates using LMM.

### Genome-wide association study (GWAS)

Before GWAS, we performed genetic ancestry inference, genotype imputation, and strict quality control on genotype data and filtered 4468 genetically unrelated preadolescents with European ancestry who passed structural image quality control (see Additional file 1 and Additional file 2: Tables S3-S6 for details). To control the confounding effects introduced by population stratification, we performed principal component analysis (PCA) on genotype and calculated the top 10 genetic principal components (Pcs) as covariates in GWAS. We identified altogether 15 regions with significantly altered CT in a single diagnosis family (internalizing: 10, externalizing: 5) and 29 regions with significantly altered SA in a comorbidity diagnosis family. To explore the genetic underpinnings of these abnormal regions of CT and SA, respectively, we therefore performed GWAS on the CT (15 regions) and SA (29 regions) using plink V2.0 [59]. Age, sex, mean CT (for regional CT) or total SA (for regional SA), top 10 genetic Pcs, and study sites were included as covariates.

Genomic risk loci were defined using the FUMA [60] online platform (version 1.3.6a). Independent significant single nucleotide polymorphisms (IndSigSNPs) were defined as variants with a *p* value < 5 × 10^−8^ and independent of other significant single nucleotide polymorphisms (SNPs) at *r*^2^ < 0.6. Lead SNPs were also identified as those independent from each other (*r*^2^ < 0.1). LD blocks for IndSigSNPs were then constructed by tagging all SNPs with MAF ≥ 0.0005 and in LD (*r*^2^ ≥ 0.6) with at least one of the IndSigSNPs. The reference panel population was European of the 1000 Genomes Project Phase 3.

On the one hand, SNPs were mapped to genes by a combination of positional, expression quantitative trait loci (eQTL) and 3-dimensional (3D) chromatin interaction mappings. Specifically, positional mapping was mapping SNPs to locus based on their physical positions. In eQTL mapping, SNPs were mapped to candidate genes according to significance criteria (*p* < 0.05) eQTL associations from Genotype-Tissue Expression (GTEx) [61] v8, the UK Brain Expression Consortium [62] (http://www.braineac.org/), the Common Mind Consortium [63], and PsychENCODE [64] (http://resource.psychencode.org). We included the major histocompatibility complex region in our FUMA analyses due to the links between the brain, psychiatric disorders, and immune system [65, 66]. Other parameters were consistent with Makowski et al. [67]. On the other hand, to combine the cumulative effects of SNPs assigned to a gene, gene-based analysis was carried out using MAGMA [68] implemented in FUMA. SNPs were mapped to genes within 50 kb upstream and downstream of the gene, a window size that has been used in previous cortical GWAS [69]. Then, the gene-based *p* values were calculated by the GWAS summary statistics of mapped SNPs, indicating the association between the gene and the GWAS phenotype. Genes significantly associated with each ROI with diagnostic effect on SA and CT were determined by Bonferroni correction (*q* = 0.05).

### Gene set enrichment analysis

To further identify the biological processes underlying regional SA and CT, we performed gene set enrichment analyses on regional SA and CT based on KEGG, GO, and GWAS catalog gene sets. All genes were set as background genes. Bonferroni correction for all analyses was applied through FUMA. Other parameters in these analyses were set as default.

### Cell type specificity analysis

To test whether genetic risk variants for regional SA and CT converge on a specific cell type, we performed cell type specificity analysis [70] using 7 single-cell RNA sequencing datasets from human brain tissue (Additional file 2: Table S7) and pre-computed MAGMA results, which builds the relationships between cell type-specific gene expression and trait–gene associations. We used Bonferroni (*q* = 0.05) correction for multiple testing per dataset to identify significantly associated cell types.

## Results

### Demographic characteristics

We consider four groups: externalizing disorders (total *N* = 883, including attention-deficit/hyperactivity disorder (*N* = 624), oppositional defiant disorder (*N* = 369), and conduct disorder (*N* = 61)), internalizing disorders (total *N* = 1961, including dysthymia (*N* = 3), major depressive disorder (*N* = 59), disruptive mood dysregulation disorder (*N* = 4), agoraphobia (*N* = 10), panic disorder (*N* = 7), specific phobia (*N* = 1379), separation anxiety disorder (*N* = 310), social anxiety disorder (*N* = 172), generalized anxiety disorder (*N* = 84), post-traumatic stress disorder (*N* = 24)), comorbid internalizing and externalizing disorders (*N* = 1054), and healthy control (*N* = 3672) groups. Demographic data for the ABCD sample is listed in Additional file 2: Tables S1 and S2. Lifetime psychiatric diagnoses were determined using KSADS based on DSM-5. To keep our healthy control group free from overlapping disorders, subjects with any recorded unspecified or specified disorders (eating disorders, alcohol use disorders, substance-related disorders, sleep problems, suicidal ideation or behavior, and homicidal ideation or behavior) were excluded from this group. A total of 1392 subjects were excluded on this basis.

### Surface area: more brain regions affected in comorbidity than in single diagnostic families

**Fig. 2:**
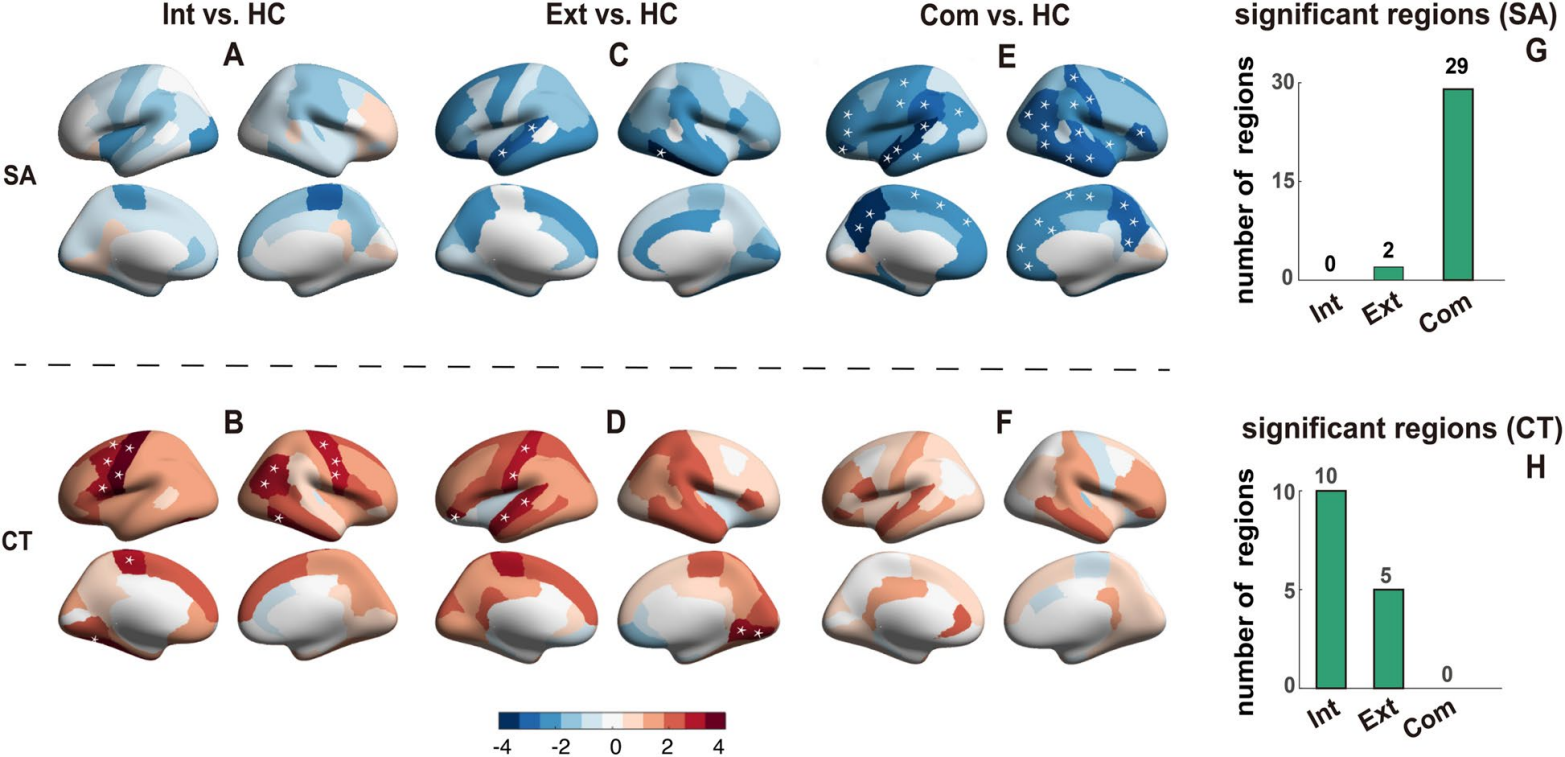
Brain regions with significant morphological alterations compared to the healthy controls in externalizing disorders group, internalizing disorders group, and the comorbidity group. The brain regions with significant morphological differences compared to the healthy controls in externalizing disorders group (**A**, **B**), internalizing disorders group (**C**, **D**), and comorbidity (**E**, **F**) in terms of cortical surface area (**A**, **C**, **E**) and cortical thickness (**B**, **D**, **F**). The color bars in **A**–**F** represent the *t* value of the regression coefficient of the group variable from the linear mixed model (LMM). The regions with * represent *p* < 0.05, FDR corrected (FDR *q* = 0.05). The number of brain regions with significant alterations for each of the three transdiagnostic groups (externalizing, internalizing, and comorbidity groups) is shown for cortical surface area (**G**) and cortical thickness (**H**). *Abbreviations*: Int, internalizing disorders; Ext, externalizing disorders; Com, comorbid between internalizing and externalizing disorders; HC, healthy control

**Fig. 3:**
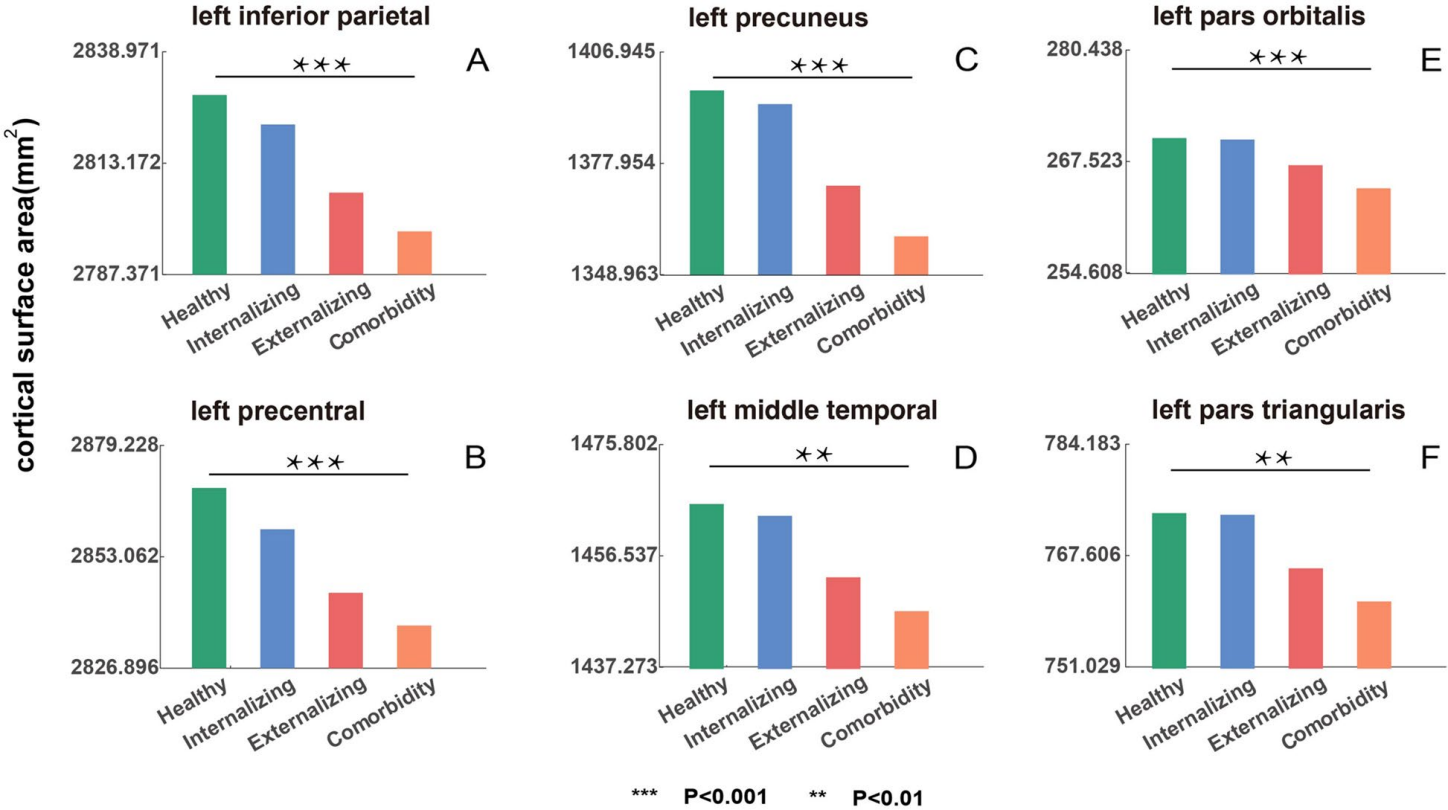
The comparison of mean cortical surface area (SA) in healthy controls, internalizing, externalizing, and comorbidity groups. The mean SA of controls, internalizing, externalizing, and comorbidity groups for regions with significant SA alterations in the comorbidity group (compared to controls). Only the top six regions with significant SA alterations in the comorbidity group were shown. Statistics of more brain regions can be found in Additional file 2: Table S8. The *y*-axis represents the mean SA. All *p* values passed FDR correction (FDR *q* = 0.05)

Children with comorbidity had pronounced SA reduction across the brain compared to the controls, while the single diagnostic groups had only a few regions with significant differences compared to the controls (Fig. 2). In particular, 29 out of 68 cortical regions demonstrated significantly lower SA in comorbid children, including the left precuneus (*t* =  − 4.23, *p* = 2.4 × 10^−5^), right middle temporal gyrus (*t* =  − 3.45, *p* = 5.6 × 10^−4^), left supramarginal (*t* =  − 3.16, *p* = 1.6 × 10^−3^) and prefrontal areas (left pars orbitalis (*t* =  − 2.96, *p* = 3.1 × 10^−3^), right pars orbitalis (*t* =  − 3.15, *p* = 1.6 × 10^−3^)), and sensory motor regions (right postcentral gyrus (*t* =  − 3.07, *p* = 2.1 × 10^−3^), left precentral gyrus (*t* =  − 2.82, *p* = 4.8 × 10^−3^)); all *p* values passed FDR correction (FDR *q* = 0.05) (see Additional file 2: Table S8). When children with externalizing disorders were compared with healthy children, only 2 temporal regions (right inferior temporal gyrus (*t* =  − 3.88, *p* = 1.1 × 10^−4^) and left superior temporal gyrus (*t* =  − 3.21, *p* = 1.4 × 10^−3^)) demonstrated a significant SA reduction, while no SA reduction was notable in the internalizing disorder group (Figs. 2 and 3). The two temporal regions with reduced SA in externalizing disorder group also showed SA reduction in the comorbid group.

The omnibus ANOVA analysis contrasting the 4 groups (internalizing, externalizing, comorbidity disorder, and healthy control groups) revealed significant changes in accordance with the above case–control results (Additional file 2: Table S9). A post hoc contrast revealed SA reduction affecting left precuneus and right pars triangularis in the comorbid group compared to the internalizing disorders and the control group, while this did not reach significance in comparison with the externalizing disorder group. Taken together, these observations indicate that the SA differences in comorbidity include those changes that are seen in externalizing disorders, at a somewhat greater magnitude; furthermore, SA differences in the comorbid group are more extensive than the minimal, insignificant deviations seen in internalizing disorders. This is also reflected in Fig. 3 in which the comorbid group is more “similar” to the externalizing disorder group than to the internalizing disorder group.

### Cortical thickness: more regions affected in single diagnostic families than comorbidity group

**Fig. 4:**
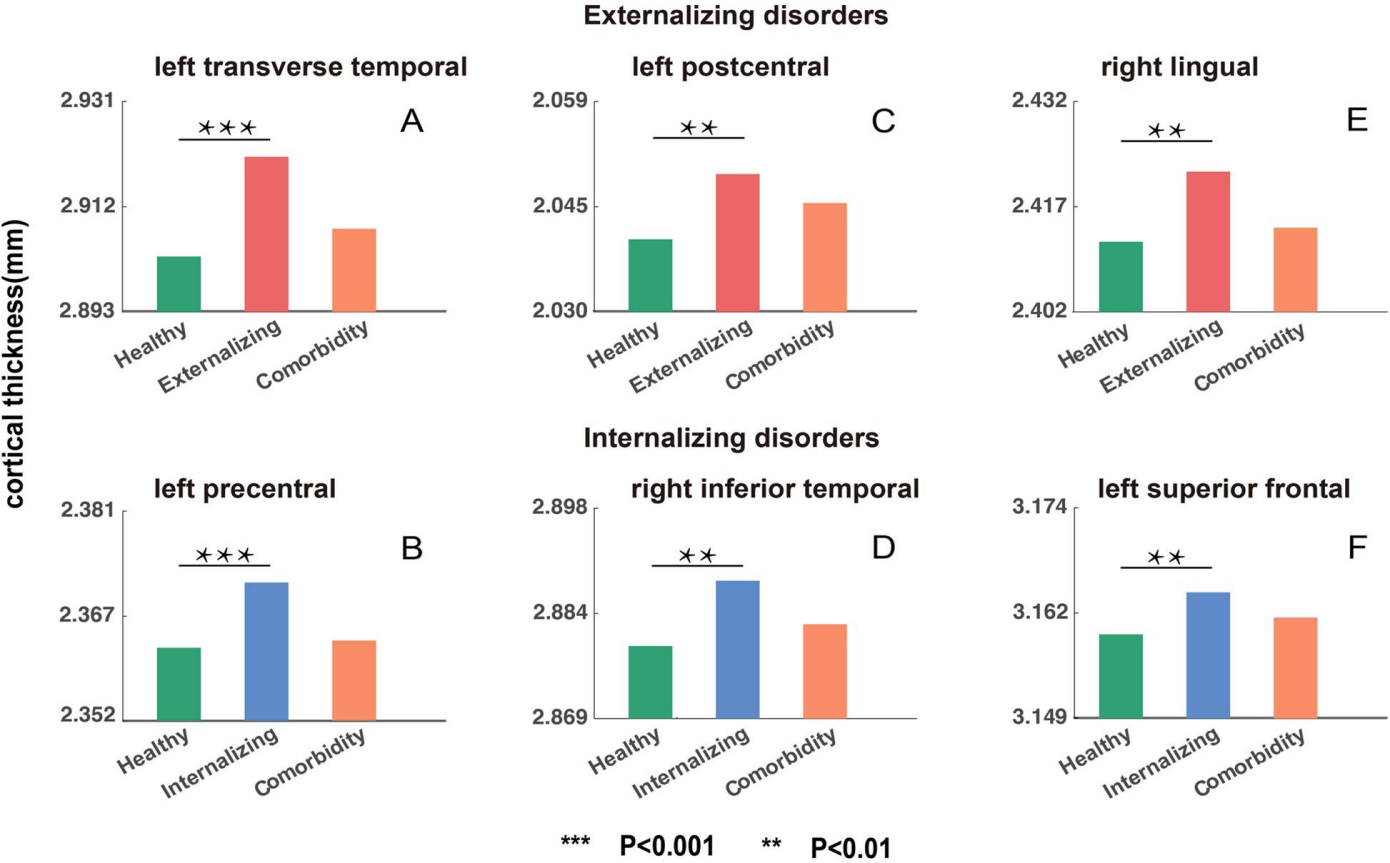
Comparison of regional cortical thickness (CT) in controls, internalizing, externalizing, and comorbidity groups. The mean CT of controls, externalizing, and comorbidity groups for regions with significant CT alterations in the externalizing group (compared to controls) (**A**, **C**, **E**). The mean CT of controls, internalizing, and comorbidity groups for the regions with significant CT alterations in the internalizing group (compared to controls) (**B**, **D**, **F)**. Only the most significant 3 regions were shown for each disorder, with the rest of the brain regions shown in Additional file 2: Table S10. The *y*-axis represents the mean CT. All *p* values passed FDR correction (FDR *q* = 0.05)

Children with either internalizing or externalizing disorders had significant alterations in CT when compared to healthy children (externalizing disorders: 5 regions, internalizing disorders: 10 regions, see Figs. 2 and 4). The comorbid group had no significant differences in CT in any of the 68 brain regions compared with the healthy children. For externalizing disorders family, auditory (left transverse temporal gyrus (*t* = 3.30, *p* = 9.9 × 10^−4^) and left superior temporal gyrus (*t* = 3.11, *p* = 1.9 × 10^−3^)), sensory-motor (left postcentral gyrus (*t* = 3.11, *p* = 1.9 × 10^−3^)), visual (right lingual (*t* = 2.89, *p* = 3.8 × 10^−3^)), and prefrontal cortex (left pars orbitalis (*t* = 2.87, *p* = 4.1 × 10^−3^)) showed significant CT differences. For internalizing disorders, sensory-motor (left precentral gyrus (*t* = 4.05, *p* = 5.1 × 10^−5^), right precentral gyrus (*t* = 3.29, *p* = 1.0 × 10^−3^) and left paracentral lobule (*t* = 2.93, *p* = 3.4 × 10^−3^)), temporal (right inferior temporal gyrus (*t* = 3.44, *p* = 5.9 × 10^−4^) and right banks of superior temporal sulcus (*t* = 2.84, *p* = 4.5 × 10^−3^)), and frontal-parietal cortices (left pars opercularis (*t* = 3.18, *p* = 1.4 × 10^−3^), left caudal middle frontal gyrus (*t* = 2.88, *p* = 4.0 × 10^−3^), right inferior parietal gyrus (*t* = 2.89, *p* = 4.0 × 10^−3^), and left superior frontal gyrus (*t* = 2.74, *p* = 6.1 × 10^−3^)) showed significant differences (Additional file 2: Table S10).

The ANOVA contrasting the 4 groups (internalizing, externalizing, comorbidity disorders, and control group) revealed significant differences in the bilateral precentral gyrus, left pars opercularis, temporal cortex (right inferior temporal gyrus and right bank of superior temporal sulcus), and right inferior parietal gyrus (Additional file 2: Table S11). A post hoc contrast revealed higher CT in bilateral paracentral lobules in children with externalizing disorders, compared to both the healthy and the comorbid groups. Children with internalizing disorders had higher CT in the left paracentral lobule and bilateral precentral gyrus compared to the other 2 groups. Taken together, these observations indicate that the CT differences in internalizing and externalizing disorders are extensive and variously distributed, but in the presence of comorbidity, these differences do not co-occur. Instead, they diminish in magnitude, leading to a pattern that is indistinguishable from healthy controls. Finally, ANOVA analysis reveals less significant changes when comparing the comorbid group to the externalizing disorder group than to the internalizing disorder group, suggesting that the comorbid group is more similar to the externalizing disorder group (Additional file 2: Table S11).

### Surface area but not cortical thickness reflects an additive effect

We found that alterations of surface area for externalizing and comorbid (internalizing and externalizing) group may reflect an additive effect. In other words, the SA differences in the comorbid state appear to be partially an aggregate of the individual differences that occur in internalizing and externalizing disorders. In contrast, CT in the comorbid group does not satisfy the expectations under an additive model, as the internalizing/externalizing group each had significant CT differences (compared to controls), but the comorbid group did not have more pronounced CT changes, as one would expect under the additive model; instead, comorbid subjects did not differ from healthy controls in their CT.

We also explored if the observed patterns of SA/CT alterations for comorbidity and single diagnostic family still hold true if we included the smaller group with “thought disorders,” a group that was not considered in the original analysis due to the small sample size. With this group included, we still observe a similar pattern of SA/CT alterations (i.e., a larger number of regional changes in the comorbidity group than single diagnostic families for SA and a reversed pattern for CT) (see Additional file 2: Table S12).

We finally explored if the observed patterns of SA/CT alterations for comorbidity and single diagnostic family simply reflect the total “burden” of psychiatric diagnoses (i.e., comorbidity within diagnostic families) or a specific combination of internalizing and externalizing disorders, by examining if there is any difference in CT/SA between children diagnosed with different number of within-family disorders. Specifically, we compared children diagnosed with 1, 2, 3, and > 3 internalizing disorders (or externalizing disorders) and did not find any difference (see Additional file 1). Based on these results, SA/CT alterations were not driven by comorbidity within a single diagnostic family, but rather by comorbidity across diagnostic families.

### Psychiatric symptom measures correlated with the surface area more significantly than cortical thickness

**Fig. 5:**
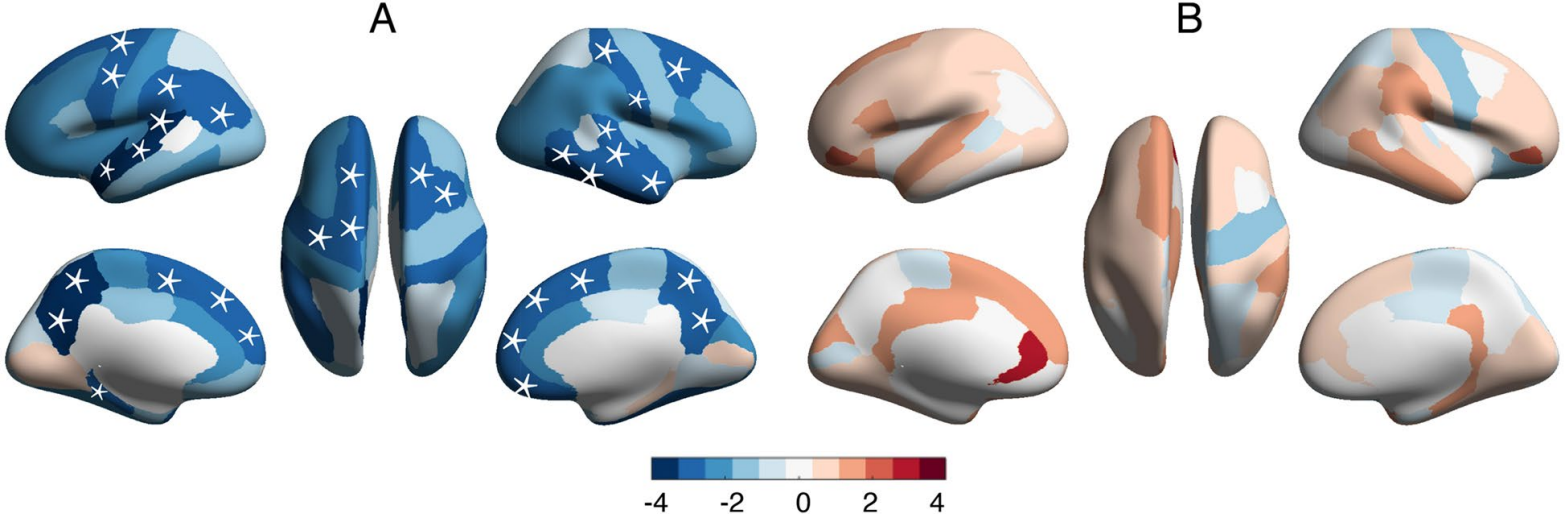
Correlation between 'p-factor' reflecting an overarching susceptibility to any mental disorder and SA and CT. Correlation between 'p-factor' and SA in comorbidity-specific regions (**A**) and correlation between p-factor and CT in externalizing-specific regions and internalizing-specific regions (**B**). The color bar represents the *t* value of the regression coefficient of the group variable from the linear mixed model (LMM). The asterisks (*) indicate *p* < 0.05, FDR correction (FDR *q* = 0.05)

We found that the p-factor, which reflects an overarching susceptibility to any mental disorder [57, 71, 72], was significantly higher in children with a notable reduction in the SA (irrespective of diagnostic status) in the same cortical regions that were prominently affected in the comorbid group (bilateral precuneus, superior and inferior temporal gyrus, and the left superior frontal gyrus; all *p* < 0.05, FDR corrected (FDR *q* = 0.05), see Fig. 5A). However, the p-factor was not correlated with the cortical thickness (Fig. 5B).

**Fig. 6:**
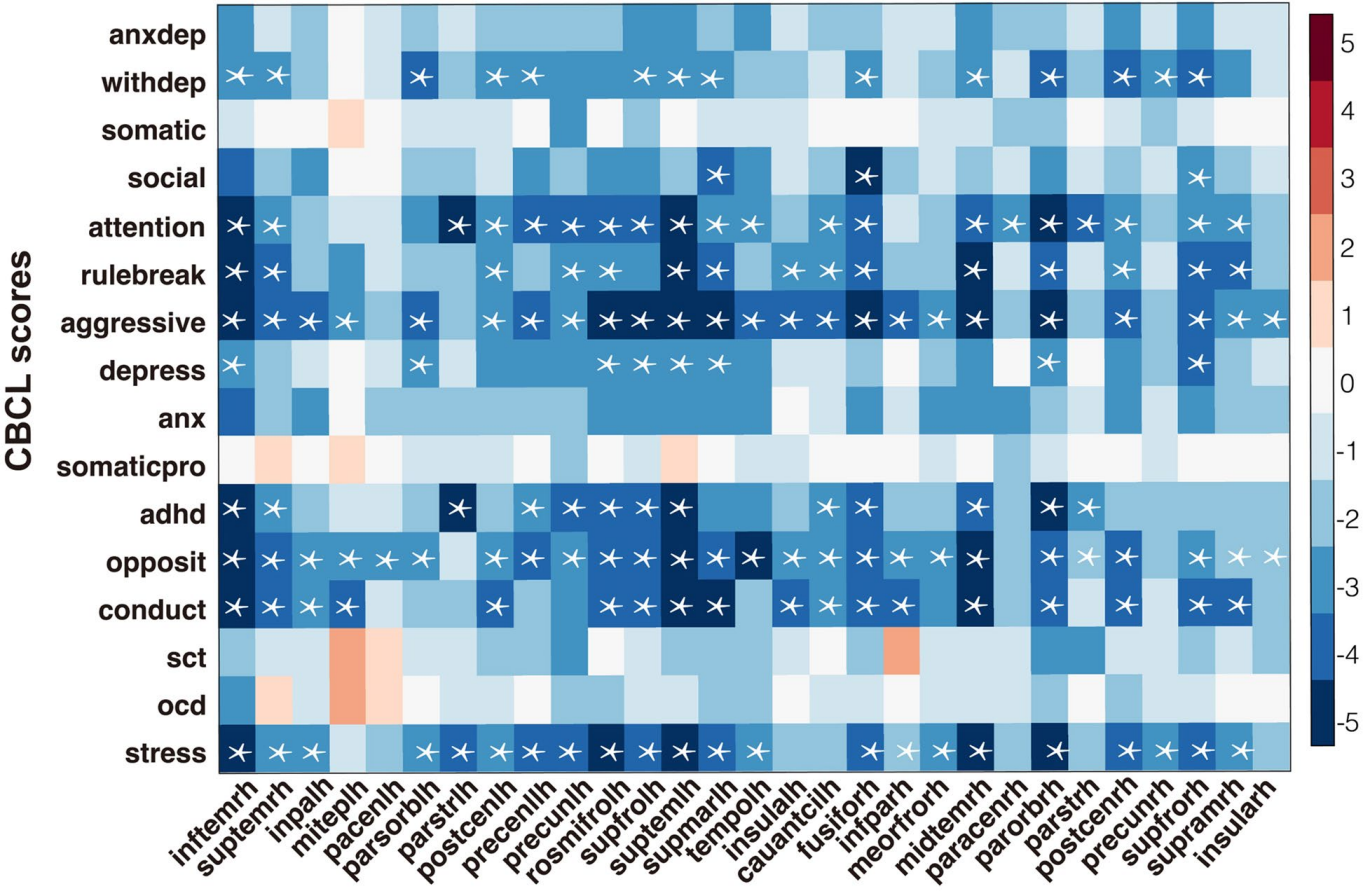
Associations between CBCL score and surface area of regions with significant alterations in patient groups. The color bar represents the *t* value of the regression coefficient from LMM. The asterisks (*) indicate *p* < 0.05, FDR correction (FDR *q* = 0.05). *Abbreviations*: AnxDep, anxious/depressed; WithDep, withdrawn/depressed; Somatic, somatic complaints; Social, social problems; Attention, attention problems; Rulebreak, rule-breaking behavior; Aggressive, aggressive behavior; Depress, depressive problems; Anx, anxiety disorders; Somaticpro, somatic problems; ADHD, attention deficit/hyperactivity problems; Opposit, oppositional defiant problems; Conduct, conduct problems; SCT, sluggish cognitive tempo; OCD, obsessive–compulsive problems; Stress, stress problems; inftemrh, right inferior temporal gyrus; suptemrh, right superior temporal gyrus; inpalh, left inferior parietal gyrus; miteplh, left middle temporal gyrus; pacenlh, left paracentral lobule; parsorblh, left pars orbitalis; parstrlh, left pars triangularis; postcenlh, left postcentral gyrus; precenllh, left precentral gyrus; precunlh, left precuneus; rosmifrolh, left rostral middle frontal gyrus; supfrolh, left superior frontal gyrus; suptemlh, left superior temporal gyrus; supmarlh, left supramarginal; tempolh, left temporal pole; insulalh, left insula; cauantcirh, right caudal anterior cingulate; fusiforh, right fusiform; infparh, right inferior parietal gyrus; meorfrorh, right medial orbito frontal gyrus; midtemrh, right middle temporal gyrus; paracenrh, right paracentral lobule; parorbrh, right pars orbitalis; parstrh, right pars triangularis; postcenrh, right postcentral gyrus; precunrh, right precuneus; supfrorh, right superior frontal gyrus; supramrh, right supramarginal; insularh, right insula

Children with lower SA in the cortical regions with a pronounced comorbidity effect also had higher CBCL externalizing problems scores (including rule-breaking behavior scores, aggressive behavior scores, oppositional defiant problems scores, and conduct problems scores) and internalizing problems scores (including withdrawn/depressed scores and depressive problems scores) with *p* < 0.05, FDR corrected (FDR *q* = 0.05) (see Fig. 6). In particular, the SA of prefrontal and temporal regions related to both externalizing and internalizing problem scores.

**Fig. 7:**
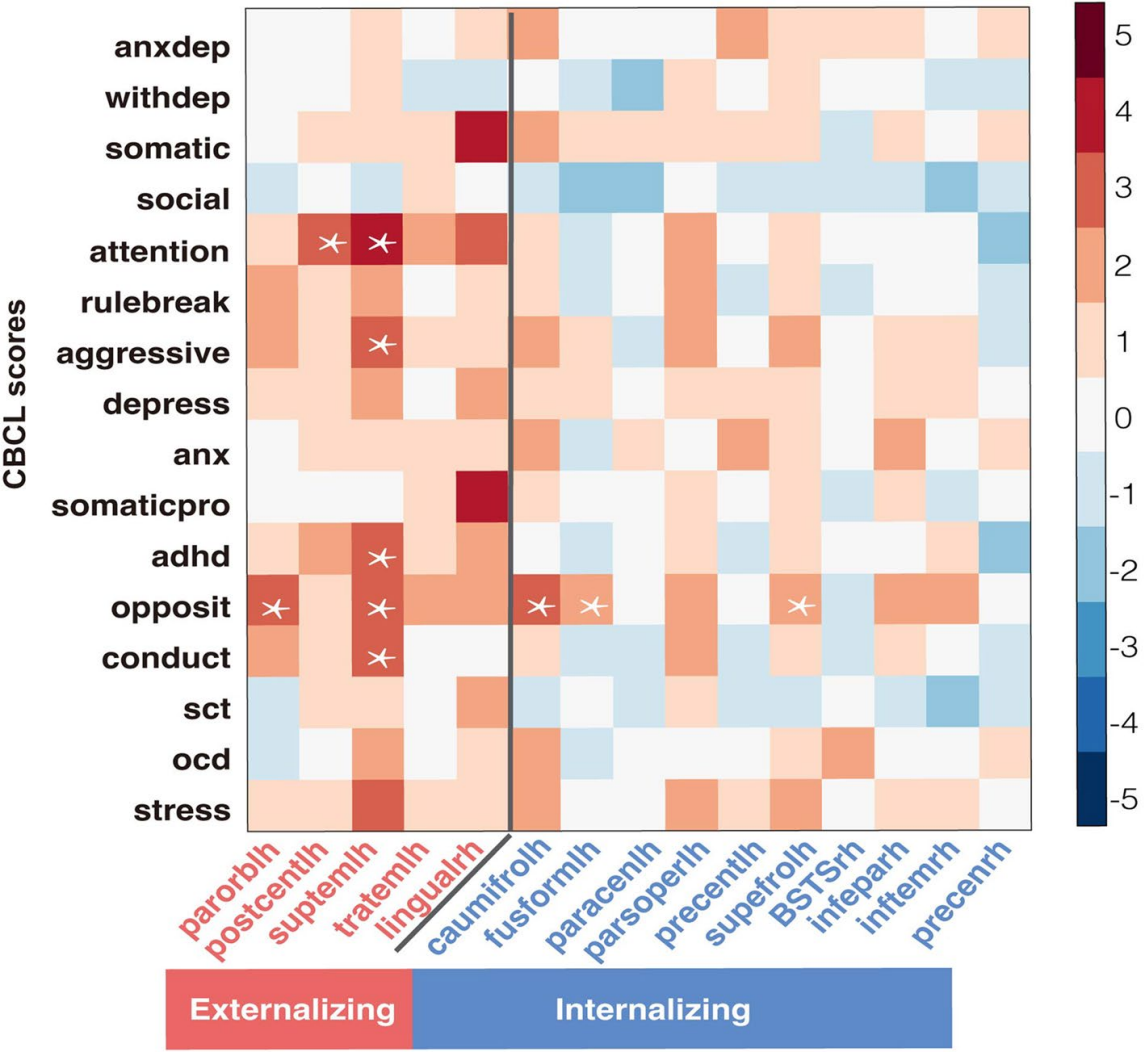
Associations between CBCL score and cortical thickness of regions with significant alterations in patient groups. The color bar represents the *t* value of the regression coefficient from LMM. The asterisks (*) indicate *p* < 0.05, FDR correction (FDR *q* = 0.05). *Abbreviations*: AnxDep, anxious/depressed; WithDep, withdrawn/depressed; Somatic, somatic complaints; Social, social problems; Attention, attention problems; Rulebreak, rule-breaking behavior; Aggressive, aggressive behavior; Depress, depressive problems; Anx, anxiety disorders; Somaticpro, somatic problems; ADHD, attention deficit/hyperactivity problems; Opposit, oppositional defiant problems; Conduct, conduct problems; SCT, sluggish cognitive tempo; OCD, obsessive–compulsive problems; Stress, stress problems; parorblh, left pars orbitalis; postcentlh, left postcentral gyrus; supetemlh, left superior temporal gyrus; lingualrh, right lingual; caumifrolh, left caudal middle frontal gyrus; fusformlh, left fusiform; paracenlh, left paracentral lobule; parsoperlh, left pars opercularis; precentlh, left precentral; supefrolh, left superior frontal gyrus; BSTSrh, right banks of superior temporal sulcus; infeparh, right inferior parietal gyrus; inftemrh, right inferior temporal gyrus; precenrh, right precentral gyrus

Children with higher CT in the cortical regions affected by externalizing disorders rather than internalizing disorders had higher CBCL externalizing problems scores (including attention scores, aggressive behavior scores, oppositional defiant problems scores, and conduct problems scores) with *p* < 0.05, FDR corrected (FDR *q* = 0.05) (see Fig. 7). In particular, the CT of temporal cortex related to externalizing problem scores.

### Distinct biological processes and cell types associated with SA and CT alterations

To understand the genetic underpinnings of the difference between SA and CT alterations across single diagnostic families (internalizing or externalizing disorders) and the comorbid group, we performed GWAS, gene set enrichment analysis, and cell type specificity analysis. We first performed GWAS for the 29 ROIs with significant SA differences and 15 ROIs with significant CT differences in the patient groups, using 4468 European-ancestry unrelated individuals. Under the classic genome-wide threshold of *p* < 5 × 10^−8^, we identified 76 genome-wide significant SNPs (after clumping) across 6 regions for CT and 139 genome-wide significant SNPs (after clumping) across 6 regions for SA (see Additional file 2: Tables S13-S14). After correcting the multiple comparisons for all 44 regions, 24 SNPs for CT and 80 SNPs survived the Bonferroni adjustment (*p* < 1.1 × 10^−9^ = 5 × 10^−8^/44, 44 is the number of all regions). Next, SNPs were mapped to genes with a combination of positional, eQTL, and 3D chromatin interaction mappings by FUMA. MAGMA gene-based association also identified several significantly associated genes in FUMA. Then, we performed enrichment analyses using all the above genes (Additional file 2: Table S15-16).

The major biological pathways that relate to SA differ from those related to CT. SA-related genes are enriched primarily in craniofacial microsomia (*p* = 1.72 × 10^−7^) and multiple system atrophy (*p* = 3.72 × 10^−5^), while CT-related genes are related to immunoglobulin light chain (AL) amyloidosis (*p* = 2.02 × 10^−5^) (Additional file 2: Tables S17-S18). Makowski et al. [67] have also found genes of the regional cortical area are significantly enriched in multiple system atrophy. Furthermore, in the gene set enrichment analysis, distributed CT alterations were associated with common genes that are related to immune-related biological processes (Additional file 2: Table S19). For example, the AHR gene was linked to CT of the left postcentral gyrus and left precentral gyrus; the TRPM8 gene was linked to CT of the right banks of the superior temporal sulcus and left precentral gyrus; the SKAP2 gene was linked to CT of the bilateral precentral gyrus. AHR is a physiological regulator of myelination and inflammatory processes in the developing central nervous system [73], implicated in psychiatric disorders like major depressive disorder [74]. TRPM8 channel augments T-cell activation and proliferation, which has been shown to be involved in mucosal sensory neurons in the regulation of innate inflammatory responses [75]. SKAP2 is a new regulator of migration and myelin sheath formation [76]. To validate our results in datasets with larger sample sizes, we also used the GWAS summary results from a previous GWAS [36] of 3144 functional and structural brain imaging phenotypes from the UK Biobank. This GWAS used 8428 subjects in the discovery dataset and 3456 subjects in the replication dataset, most of whom were of European ancestry. We also looked up the pathways associated with the same regions of CT and SA in UK Biobank using gene set enrichment analysis (see Additional file 1 and Additional file 2: Tables S20-S23).

Finally, cell-type specificity analysis for the genes associated with SA or CT alterations (Additional file 2: Tables S24-S25) uncovered further differences. SA-related genetic pathways specifically relate to the inhibitory neurons while CT-related cell types included astrocytes and oligodendrocytes. For SA, genes related to regions most affected in the comorbid group (left postcentral gyrus (*p* = 1.5 × 10^−5^) and right fusiform gyrus (*p* = 6.4 × 10^−4^)) had significant associations with In1c (inhibitory neurons). For CT, two internalizing-specific regions (left fusiform gyrus (*p* = 8.2 × 10^−4^) and right banks of superior temporal sulcus (*p* = 5.0 × 10^−3^)) showed significant associations with astrocytes. Moreover, an internalizing-specific region (left caudal middle frontal gyrus (*p* = 5.1 × 10^−4^)) showed significant associations with oligodendrocyte progenitor cells (OPC).

## Discussion

In a large preadolescent sample of 11,878 children, we studied the structural basis of comorbid expression of internalizing and externalizing disorders and report 2 major findings. First, children with comorbidity show more pronounced deviation from healthy children in cortical SA across the fronto-temporal cortex. This reduction is much more pronounced than what is seen in children with externalizing disorder who in turn show more reduction than those with internalizing disorders. The magnitude of SA reduction also tracks the severity of the “p” factor. The effect of comorbidity on the brain structure relates to comorbidity across, but not within, diagnostic families. This also indicates that within diagnostic families, common developmental origins are highly likely, and the differences in specific diagnoses do not translate to structural differences. Second, children with comorbidity show near-normal CT. This is in contrast with the aberrant differences in CT seen in children with internalizing disorders, who show more pronounced alterations compared to those with externalizing disorders. Interestingly, differences in CT did not relate to the “p” factor reflecting comorbid psychopathology. Therefore, an interesting gradient was observed in both CT and SA: the degree of differences in externalizing disorders was closer in magnitude to comorbidity than internalizing disorders for both SA and CT. This is also supported by the findings that a higher percentage of externalizing children converted to comorbidities after 2 years compared to internalizing children (Additional file 2: Table S26). Taken together, our findings indicate a specific role for the maturation of SA in the development of comorbid disorders, while the mechanistic pathways underlying individual diagnostic families likely operate via distinct aberrations in CT.

Cortical SA differences correlated significantly with the p-factor which reflects an overarching susceptibility to several mental disorders [57, 71, 72] (see Fig. 5A). This is consistent with previous studies on the ABCD cohort [21]. Furthermore, across the frontotemporal regions affected in the comorbidity group, SA significantly related to both internalizing and externalizing problem scores (Fig. 6). This again reinforced the suggestion that SA, rather than CT, underlies the emergence of a comorbid disorder pattern, and this may relate to the continuous nature of the relationship between SA and psychopathology in this age group.

The distinct patterns of structural alterations in SA and CT are in line with the fact that these two morphological measures are genetically independent [24, 26, 77]. Using cell-type specificity analysis of genes associated with regional SA and CT alterations (Additional file 2: Tables S24-S25), we parsed this further. For the left rostral middle frontal gyrus, a region with reduced SA in the comorbid group, we noted significant associations especially with In1c (inhibitory neurons). For CT, two internalizing-specific regions (left fusiform gyrus and right banks of superior temporal sulcus) showed significant associations with astrocytes and an internalizing-specific region (left caudal middle frontal gyrus) showed significant associations with OPC. Astrocytes and oligodendrocytes are the main immune cells in the brain [21, 53, 54], implicated in various psychiatric disorders in adolescence [78, 79]. Taken together, a generalized vulnerability affecting synapses and thus cortical surface area may underlie preadolescent mental disorders. In children with a higher vulnerability, SA reduction is more pronounced, and comorbid diagnostic states are expressed. An independent, immune-mediated pathway also operates in children with psychopathology, though not directly contributing to comorbidity.

We note that regions with significant CT alterations have common genetic associations that are associated with immune-related biological processes (Additional file 2: Table S19), supporting a possible role for the neuroimmune system in CT alterations underlying psychopathology. Cell-type analysis of regional CT also reveals two main kinds of brain immune cells, i.e., oligodendrocyte and astrocyte. Malfunctions of astrocyte/microglia and oligodendrocytes have been shown to affect CT in opposite ways for neurodevelopmental disorders. That is, impaired oligodendrocytes lead to insufficient myelination that results in a poorly defined gray–white boundary [43] and therefore thicker gray matter [44] during development, while astrocyte/microglia over activation [51] accompanied by more pro-inflammatory cytokines in children [80, 81] may lead to a reduction in neuronal/synaptic density [79]. Therefore, we postulate that the above two “opposite” immune-modulated processes may cause the observation that significant CT alterations occurred in a single disorder but not in the comorbidity group.

We speculate that stress level and accompanying pro-inflammatory markers increase from healthy controls to single diagnostic families to comorbidity group in a linear manner (Additional file 2: Table S27); this conjecture needs empirical confirmation (see Additional file 1 for details). One interpretation that we can make from the current data is that the development of SA and CT are affected differentially in the presence of comorbidity. In a broad sense, if we assume that a continuously distributed neurobiological mechanism underlies both internalizing and externalizing disorders, then that risk is likely reflected in the development of SA, with the most affected individuals (comorbid) displaying the most severe SA reduction and internalizing disorder being the least affected. Similarly, if we construe CT changes to reflect resilience in response to this underlying continuous dimension of risk, then higher CT may confer a higher degree of resilience, with the most resilient one displaying internalizing phenotype, and the least resilient developing notable comorbidity. The immune-related links to CT, rather than SA, provide indirect support for this developmental risk-immune-related adaptation model. We caution the readers that this is one of many possible inferences from observational data, and further empirical studies, including animal models of comorbidity, will be required to make progress.

### Limitations

Our study has several strengths as well as limitations. We examined comorbidity in one of the largest developmental neuroimaging cohorts studied to date; we used multilevel analysis linking genetic variants, cell types, and distinct morphometric variables. Nevertheless, we lacked direct measures of myelination or microglial activity to infer the mechanistic processes in CT alterations in more detail. We also lacked sufficient data to resolve the temporal relationship between brain-based metrics and the behaviors of interest. More direct evidence and verification are needed in future analysis. As the ABCD database has some recently recognized issues with the diagnosis of certain disorders—particularly ADHD and MDD—we remove the children with ADHD and MDD and performed the same analysis, i.e., estimate the difference in cortical thickness (CT) and surface area (SA) between each of three transdiagnostic groups (externalizing, internalizing, and comorbid) and the healthy children group. We still observed a similar pattern of CT/SA changes, i.e., for SA, more brain regions were affected in the comorbidity group than the single diagnostic families, while for CT, more regions were affected in single diagnostic families than the comorbidity group (see Additional file 2: Table S28). Finally, the sample size (*N* = 4468) of GWAS is relatively small compared with current large-scale GWAS (*N* > 10 k). Larger datasets with neuroimaging data from adolescent samples are needed to validate our results.

## Conclusions

Children with comorbid psychopathology had lower SA while children without comorbidity had higher CT compared to healthy children in this large cross-sectional study. Morphometric patterns in comorbid cases correspond more closely to externalizing than internalizing disorders, suggesting that externalizing problems increase secondary risks for internalizing problems at least in some patients, resulting in comorbidity. Comorbidity of mental health issues in children may be related to a specific developmental pathway affecting inhibitory neurons and synapses; this pathway may operate via aberrant surface areal expansion.

### Supplementary Information

**Additional file 1.** Supplementary information.

**Additional file 2: Table S1.** Demographics of the three transdiagnostic categories and healthy control. **Table S2.** Frequency of each single mental disorders within each group. **Table S3.** FUMA summary for main significant loci for cortical thickness. **Table S4.** FUMA summary for main significant loci for cortical surface area. **Table S5.** Genes associated with cortical thickness in single internalizing-specific and externalizing-specific regions. **Table S6.** Genes associated with cortical surface area in comorbid-specific regions. **Table S7.** Single-cell RNA sequencingdatasets. **Table S8.** Results of case control for SA between three diagnostic families and healthy controls. **Table S9.**Results of ANOVA and Turkey Test for SA. **Table S10.** Results of case control for CT between three diagnostic families and healthy controls. **Table S11.** Results of ANOVA and Turkey Test for CT. **Table S12.** Results considering thought disorders. **Table S13.** FUMA summary for main 76 significant loci for cortical thickness. **Table S14.** FUMA summary for main 139 significant loci for cortical surface area. **Table S15.** Genes associated with cortical surface area in comorbid-specific regions. **Table S16.** Genes associated with cortical thickness in single internalizing-specific and externalizing-specific regions.** Table S17.** Significantly enriched terms/phenotypes on genes associated with cortical surface area in comorbid-specific regions. **Table S18.** Significantly enriched terms/phenotypes on genes associated with cortical thickness in single internalizing-specific and externalizing-specific regions. **Table S19.** Different brain regions share genes involved in biological processes. **Table S20.** Genes associated with cortical surface area in comorbid-specific regions. **Table S21.** Genes associated with cortical thickness in single internalizing-specific and externalizing-specific regions. **Table S22.** Significantly enriched terms/phenotypes on genes associated with cortical surface area in comorbid-specific regions. **Table S23.** Significantly enriched terms/phenotypes on genes associated with cortical thickness in single internalizing-specific and externalizing-specific regions. **Table S24.** Cell type specificity analysis for cortical surface area. **Table S25.** Cell type specificity analysis for cortical thickness. **Table S26.** The conversion rate between single diagnostic families and comorbidity group. **Table S27.** Results of case control for stress score between three diagnostic families and healthy controls. **Table S28.** Results considering ADHD and MDD.

## Data Availability

The data from the ABCD study is available by request (see https://abcdstudy.org/scientists/data-sharing/).

## Abbreviations

3D: Three-dimensional
ABCD: Adolescent Brain Cognitive Development
ADHD: Attention deficit hyperactivity disorder
Aggressive: Aggressive behavior
AGP: Agoraphobia
ANOVA: Analysis of variance
Anx: Anxiety disorders
AnxDep: Anxious/depressed
Attention: Attention problems
BMI: Body max index
BSTSrh: Right banks of the superior temporal sulcus
cauantcirh: Right caudal anterior cingulate
caumifrolh: Left caudal middle frontal gyrus
CBCL: Child Behavior Checklist
CD: Conduct disorder
Com: Comorbid between internalizing and externalizing disorders
Conduct: Conduct problems
CT: Cortical thickness
CTSA: Cell type specificity analysis
DEL: Delusions
Depress: Depressive problems
DMDD: Disruptive mood dysregulation disorder
DSM-5: Fifth Diagnostic and Statistical Manual of Mental Disorders
DYS: Dysthymia
eQTL: Expression quantitative trait loci
EXT: Externalized disorder
FDR: False discovery rate
fusformlh: Left fusiform
fusiforh: Right fusiform
GAD: Generalized anxiety disorder
GSEA: Gene set enrichment analysis
GTEx: Genotype-Tissue Expression
GWAS: Genome-wide association study
HC: Healthy control
IL-1β: Interleukin-1 beta
IL-6: Interleukin-6
IndSigSNPs: Independent significant single nucleotide polymorphisms
infeparh: Right inferior parietal gyrus
infparh: Right inferior parietal gyrus
inftemrh: Right inferior temporal gyrus
inpalh: Left inferior parietal gyrus
insulalh: Left insula
insularh: Right insula
INT: Internalized disorder
KSADS: Kiddie Schedule for Affective Disorders and Schizophrenia
lingualrh: Right lingual
LMM: Linear mixed models
MDD: Major depressive disorder
meorfrorh: Right medial orbito frontal gyrus
midtemrh: Right middle temporal gyrus
miteplh: Left middle temporal gyrus
OCD: Obsessive-compulsive problems
ODD: Oppositional defiant disorder
OPC: Oligodendrocyte progenitor cell
Opposit: Oppositional defiant problems
pacenlh: Left paracentral lobule
PAN: Panic disorder
paracenlh: Left paracentral lobule
paracenrh: Right paracentral lobule
parorblh: Left pars orbitalis
parorbrh: Right pars orbitalis
parsoperlh: Left pars opercularis
parsorblh: Left pars orbitalis
parstrh: Right pars triangularis
parstrlh: Left pars triangularis
PCA: Principal component analysis
Pcs: Principal components
p-factor: Psychopathology factor
PFC: Prefrontal cortex
postcenlh: Left postcentral gyrus
postcenrh: Right postcentral gyrus
postcentlh: Left postcentral gyrus
precenllh: Left precentral gyrus
precenrh: Right precentral gyrus
precentlh: Left precentral
precunlh: Left precuneus
precunrh: Right precuneus
PTSD: Post-traumatic stress disorder
rosmifrolh: Left rostral middle frontal gyrus
Rulebreak: Rule-breaking behavior
SA: Cortical surface area
SCT: Sluggish cognitive tempo
SEP: Separation anxiety disorder
SNPs: Single nucleotide polymorphisms
SOC: Social anxiety disorder
Social: Social problems
Somatic: Somatic complaints
Somaticpro: Somatic problems
SPH: Specific phobia
Stress: Stress problems
supefrolh: Left superior frontal gyrus
supetemlh: Left superior temporal gyrus
supfrolh: Left superior frontal gyrus
supfrorh: Right superior frontal gyrus
supmarlh: Left supramarginal
supramrh: Right supramarginal
suptemlh: Left superior temporal gyrus
Suptemrh: Right superior temporal gyrus
tempolh: Left temporal pole
THO: Thought disorder
WithDep: Withdrawn/depressed
