# Supplementary material for: Neurodevelopmental risk and adaptation as a model for comorbidity among internalizing and externalizing disorders: genomics and cell-specific expression enriched morphometric study
Source: BMC Med. 2023 Aug 4;21:291. doi: 10.1186/s12916-023-02920-9 (PMC10403847; doi:10.1186/s12916-023-02920-9)
Supplement: Supplementary file 1 — Additional file 1. Supplementary information. [file 12916_2023_2920_MOESM1_ESM.docx]

**Additional File1 for**

**Neurodevelopmental risk and adaptation as a model for comorbidity among internalizing and externalizing disorders: genomics and cell-specific expression enriched morphometric study**

**Supplemental methods**

**Genetic data**

Saliva samples were genotyped using the Smokescreen array [33]. We removed individuals with missing genetics or in plate 461 and SNPs (single nucleotide polymorphisms) with > 5% minor allele frequency or < 20% of the sample missing. In order to determine the genetic ancestry of ABCD samples, we firstly trained random forest classifier model on HCP (Human Connectome Project) genotypes and 1KG Project data, which could assign samples to one of the five ancestries [African (AFR), American (AMR), East Asian (EAS), European (EUR) and South Asian (SAS)] (details can be found at https://github.com/Annefeng/PBK-QC-pipeline). Then we computed genetic principal components (PCs) across 302,901 high-quality autosomal SNPs in the combined ABCD and the Phase 3 of 1000 Genomes (1KG) Project reference samples. Finally we used this random forest classifier model to assign genetic ancestries to ABCD. Imputation was performed using the Michigan Imputation Server [34] with hrc.r1.1.2016 reference panel and Eagle v2.3 phasing. Best guess conversion at a threshold of 0.9 was used to convert dosage files to plink binary PED files. Post- imputation quality control excluded individuals with >10% missing rate and SNPs with imputation info score <0.3, >5% missing rate, MAF (Minor Allele Frequency) <1%, or out of Hardy-Weinberg equilibrium violation (p>10-6), yielding 4,326,912 SNPs. We performed genetic Principal Component Analysis on genetically unrelated (PI_HAT<0.2) European individuals and derived first ten genetic principal components to correct for population stratification.

### P factor

A general psychopathology factor and three sub-factors, externalized disorder, internalized disorder and thought disorder, were modeled using the parent-rated K-SADS-5. Based on a previous literature that also used data from ABCD study [35], a hierarchy model including externalizing (ADHD, ODD, CD), internalizing (MDD, GAD, PTSD, PD, SEP, SAD), and thought (hallucinations, delusions, OCD, BP) disorder pathology, as well as a p factor using confirmatory factor analysis (R v4.0, cfa function of lavaan package). The analysis was based on the whole sample (N = 11,878) and the final factor scores were used in association analyses.

**Supplemental Results**

### Conversion rate between single diagnostic families and comorbidity group

We used 2-year follow-up ABCD data (474 externalizing disorders, 982 internalizing disorders, 541 comorbidities between internalizing and externalizing disorders) to evaluate the conversion rate between single diagnostic families (internalizing disorders and externalizing disorders) and comorbidity group. The number of each diagnostic family after two years divided by the number of each diagnostic family at baseline is the conversion rate.

### The influence of thought disorders and within-diagnostic family comorbidity

We excluded children with thought disorders due to the small sample size. To explore if the observed patterns of SA/CT alterations (for single diagostic famile and comorbidity) still hold for thought disorders, we compared the following 3 groups and the healthy children: 1. Children with thought disorders; 2. Children with comorbidity between externalizing and thought disorders and 3. Children with comorbidity between internalizing and thought disorders. We found similar patterns of SA/CT alterations for thought disorders. That is, comorbidity (group 2 and 3) has more affected regions in SA than single diagnostic family (group 1), while for CT, opposite patterns were observed (see Additional file 2: Table S12). This indicate that the observed patterns still hold if thought disorders was involved.

We also explored if the observed patterns of SA/CT alterations for comorbidity simply reflect the total ‘burden’ of psychiatric diagnoses (comorbidity within diagnostic families), rather than a specific combination of externalizing and externalizing disorder (comorbidity across different diagnostic families). We compared SA/CT of children within a single diagnostic family, but with different number of diagnosis (e.g., children diagnosed with 1, 2, 3, and >3 internalizing disorders) with controls and found that children diagnosed with different number of internalizing disorders did not show significant difference in SA/CT in any of the 68 brain regions. This results indicate that only comorbidity across different diagnostic families lead to the observed pattern of SA/CT alterations.

**The influence of ADHD and MDD**

Because the ABCD database has some recently recognized issues with the diagnosis of certain disorders- particularly ADHD and MDD, we removed the children with ADHD and MDD and employed linear mixed models (LMM) to estimate the difference in cortical thickness (CT) and surface area (SA) between each of three transdiagnostic groups (externalizing, internalizing and comorbid) and the healthy children group. The results are still similar to those without removing ADHD and MDDs, i.e., for SA, more brain regions were affected in comorbidity than single diagnostic families, while for CT, more regions affected in single diagnostic families than the comorbidity group. It should be noted that less brain regions are identified due to the reduction in sample size. Following is the details:

For SA, children with comorbidity had pronounced SA reductions in right precuneus (t=-2.54, p=0.01) and left precuneus (t=-2.39, p=0.02) compared to the controls (FDR corrected), while the single diagnostic groups had no significant regions (Additional file 2: Table S28). For CT, children with internalizing disorders had significant alterations in CT in 9 regions involving frontal, parietal and temporal cortex (Additional file 2: Table S28), which overlaps with the original analysis. Children with externalizing disorders had no significant regions due to small sample size. The comorbid group had no significant alterations.

**Pathways associated with the same regions in the UK Biobank**

We downloaded the GWAS summary statistics of the same regions (15 regions of CT and 29 regions of SA) as those in the manuscript from one previously published large-scale (22,138, updated version) UK Biobank brain imaging GWAS[36]. We did functional annotation and mapping (Additional file 2: Table S20-21) on the GWAS summary data using the same procedures as those in the manuscript. Then we performed gene set enrichment analysis separately for genes mapped from SNPs associated with regions of CT and SA (Additional file 2: Table S22-23). For regions of CT altered in single diagnosis groups, although there were few overlapping genes, there were some similarities between the pattern of enriched pathways in UK Biobank (Additional file 2: Table S23) and that in ABCD (Additional file 2: Table S18). First, they both included mental disorders or neurodegenerative disorders. Enriched pathways in UK Biobank included ‘Alcohol use disorder’, ‘Parkinson's disease’ and ‘Neuroticism’ while those in ABCD included Schizophrenia and Autism spectrum disorder. Second, they both included traits relevant to lung diseases or blood pressure. ‘Idiopathic pulmonary fibrosis’ and ‘Systolic blood pressure’ were enriched in UK Biobank while ‘Diastolic blood pressure x smoking status (current vs non-current) interaction’ and ‘Chronic obstructive pulmonary disease or high blood pressure’. For regions of SA altered in comorbidity diagnosis groups, there were few overlapping genes and no overlapping enriched pathways between UK Biobank and ABCD. In summary, considering the UK Biobank is an adult cohort with much larger sample size than that in ABCD, it is unsurprising that there were few overlapping genes and pathways between two cohorts. Nevertheless, there were still some similarities in the pattern of pathways for regions of CT altered in single diagnosis groups.

**GWAS accounting for familiar relatedness**

We used fastGWA [37] implemented in Genome-wide Complex Trait Analysis (GCTA) [38] to perform GWAS, a resource-efficient tool for Mixed Linear Model Association (MLMA) test, which takes into account familial relatedness using a sparse genetic relatedness matrix (GRM). The GWAS was performed on ABCD participants with predominantly European ancestry (N=5604). We firstly derived a full dense GRM for European ancestry participants using --make-grm flag. Then we created a sparse GRM using --make-bK-sparse flag with a threshold of <0.05, setting any relatedness between participants to 0 if their estimated relatedness is <0.05. The covariates adjusted in GWAS were consistent with those in the main text.

We identified 15 and 83 genome-wide significant SNPs for CT and SA (Additional file 2: Table S3-4), respectively, which were much fewer than those identified in the main text. For CT, left precentral gyrus, which had the greatest number of significant SNPs in the main text, did not had any significantly associated SNPs here. Right STS only had 6 significant SNPs. Therefore, the issue that some brain regions disproportionately driving the results did not exist for CT when accounting for familial relatedness. For SA, left precentral gyrus was still the region with greatest number (n=73) of significant SNPs.

Using the same SNP mapping strategy as that in the main text, we mapped genome-wide significant SNPs to 19 genes and 149 genes for CT and SA (Additional file 2: Table S5-6), respectively. We performed gene set enrichment analyses on mapped genes for CT and SA separately, using the same parameters as those in the main text. For CT, genes were enriched in ‘Diastolic blood pressure x smoking status interaction’, which was quite similar with that in the main text. For SA, genes were enriched in ‘Craniofacial microsomia’ and ‘Lipoprotein (a) levels’, which were also quite similar with that in the main text. However, this enrichment pattern was inevitably driven by left precentral gyrus while other regions with smaller number of significant SNPs made little contribution.

In fact, methods accounting for familial relatedness like fastGWA could include only about 1000 more participants in ABCD than that in the main text, which excluded genetically related individuals. This could not boost the sample size to a much extent, which restricted the power of GWAS. Therefore, it is unsurprising that the enrichment pattern for SA was still driven by left precentral gyrus, which had most significant SNPs. Nevertheless, to increase the sample size and GWAS power, we could incorporate more adolescent brain imaging and genotype datasets to do meta-analyses, which would be our future research focus.

**Possible immune-related processes underlying CT alteration**

We postulate two "opposite" immune-modulated processes may cause the observation that significant CT alterations occurred in single disorder but not in comorbidity group:

1. Oligodendrocytes: oligodendrocytes are responsible for myelination [39]. For children with depression and ADHD, oligodendrocytes have been shown be impaired or reduced in number [40,41] which was associated with more pro-inflammatory cytokines [42], leading to weak myelination. Recently it has been shown that voxels with less myelin near the gray–white matter boundary will appear darker in children than adults, which shifts the apparent gray–white boundary deeper into the white matter [43] and thus leads to thicker gray matter [44]. Therefore, children with internalizing/externalizing disorder may have reduced intracortical myelination thus thicker CT than controls.

2. Astrocyte/microglia: astrocyte/microglia plays a physiological role in neurodevelopmental disorders like ADHD [45,46], in which proinflammatory cytokines are generally increased that over-activate astrocytes and microglia [47]. Overactivated glial cells play an important part in reducing synaptic [48] and neuronal counts [49] during maturation. As stress has been shown to be associated with increased pro-inflammatory cytokines and inflammation for children [50]. Higher stress-related inflammatory activity in the comorbid group (Additional file 2: Table S27) may exacerbate astrocyte/microglial activation and thus contribute to synaptic/neuronal elimination [51, 52] thus decreasing the CT.

In sum, oligodendrocytes dysfunctions and astrocytes/microglia overactivation may lead to thick and thin CT, respectively, all related to increased level of pro-inflammatory markers. As we showed that stress level increases from controls to single diagnostic families to comorbidity group, and considering that stress level also correlated positively with the pro-inflammatory markers (interleukin (IL)-1β and IL-6) in psychiatric disorder [53,54], pro-inflammatory markers thus are also expected to increase from controls to single diagnostic families to comorbidity group. Therefore for single diagnostic families with increased pro-inflammatory markers, oligodendrocyte impairments play a major role and leads to thick cortical thickness. For the comorbidity group in which stress and pro-inflammatory factor are further increased, astrocytes/microglia overactivation is present, leading to reduced synaptic and neuronal counts and likely thinner cortical thickness, counteracting the thick thickness seen in single diagnostic families. Thus comorbidity group does not show significant CT alterations.
